# Ultrasound-guided percutaneous renal biopsy at a university hospital: retrospective analysis of success and complication rates

**DOI:** 10.1590/0100-3984.2020.0064

**Published:** 2021

**Authors:** Orlando Vieira Gomes, Bianca Alencar Dias de Almeida, Leonardo Fernandes e Santana, Mateus de Sousa Rodrigues, Guilherme Bruno Pires Marques Locio, Carla Santos Araújo, Carlos Henrique de Sousa Rosas, Marcos Duarte Guimarães

**Affiliations:** 1 Universidade Federal do Vale do São Francisco (Univasf), Petrolina, PE, Brazil.; 2 Hospital Universitário da Universidade Federal do Vale do São Francisco (HU-Univasf), Petrolina, PE, Brazil.; 3 A.C.Camargo Cancer Center, São Paulo, SP, Brazil.

**Keywords:** Biopsy, needle/methods, Kidney diseases/epidemiology, Kidney glomerulus/pathology, Ultrasonography, interventional, Biópsia por agulha/métodos, Nefropatias/epidemiologia, Glomérulos renais/patologia, Ultrassonografia

## Abstract

**Objective:**

To evaluate the success and complication rates of ultrasound-guided renal biopsy at a tertiary care hospital.

**Materials and Methods:**

This was a retrospective analysis of 97 ultrasound-guided renal biopsies, all performed by the same radiologist, between 1 March, 2017 and 31 October, 2019.

**Results:**

Of the 97 biopsies evaluated, 87 had a definitive pathological diagnosis. In five cases (5.4%), the biopsy results were inconclusive and a second procedure was required. In seven procedures (7.6%), there were complications, all of which were properly resolved.

**Conclusion:**

Ultrasound-guided renal biopsy has proven to be a safe, effective method for the diagnosis of nephropathies, with high success rates.

## Abstract (pt)

**Objetivo:**

Avaliar taxas de sucesso e de complicações de biópsias renais guiadas por ultrassonografia em um hospital terciário.

**Materiais e Métodos:**

Análise retrospectiva de 97 biópsias renais realizadas entre 1º de março de 2017 e 31 de outubro de 2019, guiadas por ultrassonografia e executadas por um único médico radiologista.

**Resultados:**

Oitenta e sete biópsias apresentaram diagnóstico anatomopatológico definitivo. Cinco biópsias foram inconclusivas (5,4%) e precisaram de um segundo procedimento. Houve complicações em sete procedimentos (7,6%), todas devidamente solucionadas.

**Conclusão:**

A biópsia renal guiada por ultrassonografia demonstrou ser um método efetivo e seguro para o diagnóstico de nefropatias, com elevada taxa de sucesso em sua execução.

## INTRODUCTION

The use of renal biopsy as a diagnostic tool has enabled a greater understanding of the pathogenesis of several kidney diseases, from glomerulopathies to focal lesions, such as nodules, tumors, and complex cysts^(1)^. Its use has grown, especially in the last 50 years, aiming mainly to stratify risks and avoid unnecessary ablative surgeries and therapies, as well as to clarify the diagnosis and prognosis of glomerular diseases^(2,3)^. In experienced hands, the procedure is associated with low complication rates (< 5%), which has made it a routine instrument for clinical evaluation^(3,4)^.

The most frequently used imaging methods for guided procedures are ultrasound and computed tomography (CT). The choice of methods depends on many factors, including equipment availability, patient age, and the risks of exposure to ionizing radiation, as well as the physician and patient preferences. The advantages of ultrasound are that it allows real-time multiplanar imaging, can be used at the bedside, is versatile, does not use ionizing radiation, and is more affordable than are traditional methods, such as surgical biopsy. One disadvantage of ultrasound is that it is operator-dependent^(1,5)^.

Image-guided percutaneous renal biopsy may be used in order to diagnose suspected malignant lesions, defining whether they are of primary or secondary origin, as well as being used as a tool for the differential diagnosis of parenchymal nephropathies^(3,4)^. Currently, the use of 16-gauge needles is recommended for the procedure. The needle gauge has a direct influence on the number of clusters obtained, as well as on complication rates. The outer diameter of a 16-gauge needle is 700 µm, whereas that of an 18-gauge needle is 350 µm. When considering the glomerular diameter in a normal adult (250 µm), the 18-gauge needle may not be of a caliber sufficient to obtain adequate samples. However, the use of a larger gauge needle (e.g., a 14-gauge needle, which has an outer diameter of 1,000 µm) is associated with a higher risk of complications without obtaining a higher number of glomeruli. Therefore, in order to strike a balance between lower complication rates and higher success rates in the collection of adequate samples, the 16-gauge needle is the most attractive choice^(6)^.

The most common complication of image-guided percutaneous renal biopsy is hemorrhage. Although small hematomas can be detected in up to 91% of the procedures^(7)^, clinically significant hemorrhage is rare. Macroscopic hematuria and hematomas have been observed, respectively, in 2-4% and approximately 20% of patients. Most of these incidents evolve to spontaneous resolution and are treated conservatively. The most feared complication is massive hemorrhaging, which occurs in 1-2% of procedures, especially in elderly patients, in patients with renal dysfunction (glomerular filtration rate < 30 mL/min/1.73 m^2^), and in patients with uncontrolled hypertension^(3)^. Other, less common, complications include urinary clots (in 0.3% of procedures), tumor spread through needle-track seeding (in < 0.01%), and intra-abdominal organ perforation, which is extremely rare^(3,7)^.

The present study aims to evaluate the success rates and complication rates of ultrasound-guided renal biopsies in patients with parenchymal nephropathies performed at a tertiary hospital. We also describe the pathological findings.

## MATERIALS AND METHODS

This was a retrospective study in which we analyzed 97 renal biopsies performed between March 1, 2017 and October 31, 2019 at the University Hospital of the Federal University of Vale do São Francisco, located in the city of Petrolina, in the Brazilian state of Pernambuco.

All of the biopsies analyzed in this study were performed in patients being followed at the glomerulopathy outpatient clinic of the hospital or in patients who developed acute kidney injury due to undefined causes during hospitalization. Patients previously submitted to renal biopsy were excluded, as were those in whom biopsy was performed to investigate a suspected malignant lesion.

Data were collected from renal biopsy reports and from the hospital medical records department. The study was approved by the Human Research Ethics Committee of the Federal University of Vale do São Francisco (Reference no 83840317.4.0000.5196). Because the study involved only a review of electronic medical records, the requirement for written informed consent was waived. The procedures and materials cited in this project are compliant with the Declaration of Helsinki.

The main indications for renal biopsy in this study were as follows: urinary anomalies, defined as hematuria or non-nephrotic proteinuria; nephrotic syndrome; nephritic syndrome; acute kidney injury/rapidly progressive glomerulonephritis; and chronic kidney disease. After the exclusion criteria had been applied, the sample comprised 92 renal biopsies. We studied variables related to the techniques employed in and the complication rates related to those biopsies. In addition, we analyzed the pathology findings for the biopsies, as determined by a renal pathologist.

All procedures were performed by the same radiologist, who had more than five years of experience, using a 16-gauge needle (Speed Cut; Gallini S.r.l., Mantova, Italy), and were guided by ultrasound with a Logiq P5 system (GE Healthcare, Wauwatosa, WI, USA). For the biopsy of native kidneys, 16- or 18-gauge needles are typically used^(3)^.

**Figure 1.**
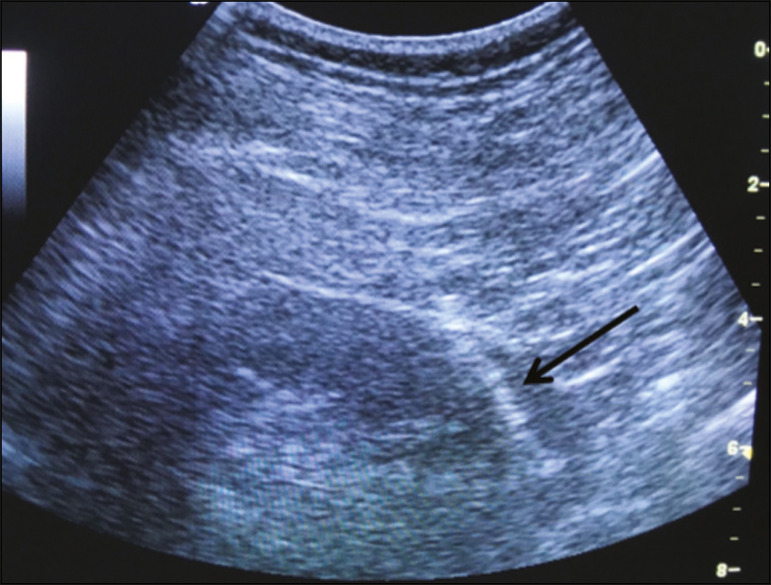
Ultrasound image obtained during percutaneous renal biopsy. Arrow showing the needle, characterized by linear hyperechoic imaging, entering the renal parenchyma at the lower pole.

**Figure 2.**
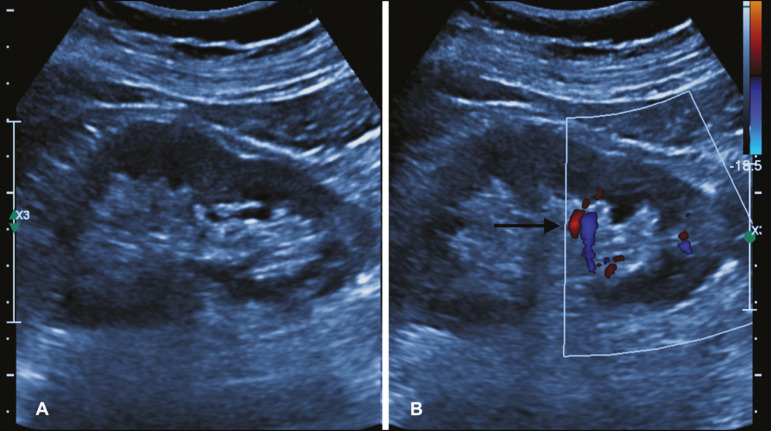
**A:** Follow-up ultrasound performed 60 min after the biopsy, showing no sign of free fluid surrounding the left kidney. **B:** Ultrasound with color Doppler showing normal renal vascularization without signs of bleeding (arrow).

In accordance with the institutional protocol at our hospital, the ultrasound-guided renal biopsy procedure involved the following steps: a) placing the patient in the prone or lateral position; b) asepsis and antisepsis of the procedure site; c) local anesthesia with lidocaine 2%; d) ultrasound study to identify the region to be biopsied (posterior lower pole of the kidney); e) choice of the needle path and marking of the needle entry site on the skin surface; f) introduction of the needle under real-time ultrasound guidance (Figure 1); g) collection of two to three fragments by core biopsy; h) packing fragments in specific material for analysis; i) post-procedure ultrasound monitoring (Figure 2); j) four hours of absolute patient bed rest, during which blood pressure and heart rate are monitored every 15 min for the first 2 h, every hour for the subsequent 4 h, every 2 h for the subsequent 6 h, and every 4 h for the subsequent 12 h; k) if there are no complications, discharge after a 24-h observation period.

**Figure 3.**
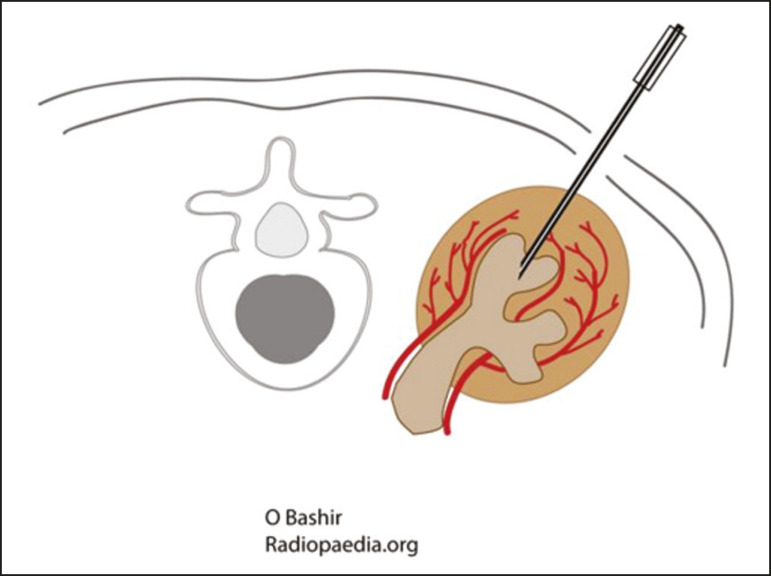
Schematic drawing showing the avascular plane of Brodel, an area favorable for performing renal percutaneous procedures such as nephrostomy and parenchymal biopsy. Figure extracted from Radiopaedia, authored by Bashir^(8)^, with permission of the author.

**Figure 4.**
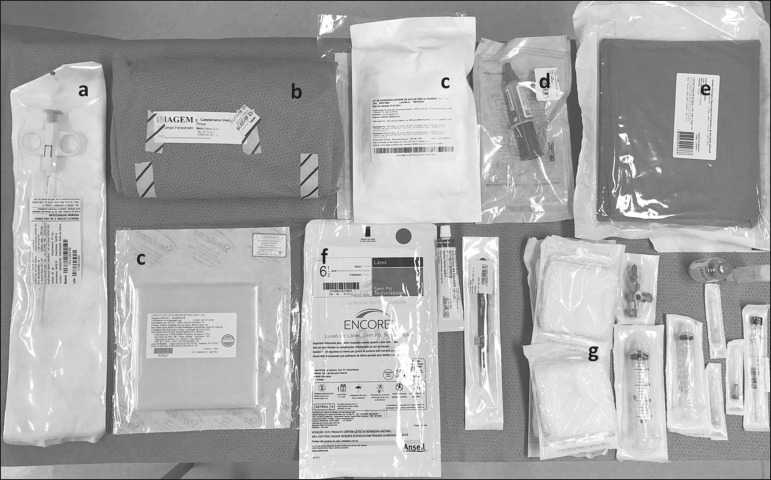
Table with materials for renal biopsy: needle for semi-automated core biopsy (**a**); sterile kit for small procedures (**b**); sterile material for covering the keyboard and ultrasound transducer (**c**); sterile gel (**d**); surgical/sterile field (**e**); sterile gloves (**f**); and auxiliary materials, including a scalpel, gauze, syringes, and anesthetic (**g**).

Biopsy was performed in the lower posterior portion of the kidney due to the presence of the area called “the avascular plane of Brodel”-the posterior portion of the renal parenchyma between the anterior and posterior branches of the renal artery (Figure 3)-in which there is a relative lack of blood vessels^(8)^. The procedure was performed with a sterile technique (Figure 4) and with the aid of color Doppler, which allows the characterization of intrarenal vascular structures, reducing the risks of hemorrhagic complications. In the procedure room, samples for optical microscopy and immunofluorescence analysis were fixed in formalin and packed in Michel’s medium, respectively, and sent to the microscopy laboratory.

In accordance with the protocol adopted by our department, all renal biopsies were performed only after laboratory confirmation of conditions favorable to the procedure (duration of bleeding, prothrombin time, and active partial thromboplastin time all within normal values; platelet count ≥ 50,000/mm^3^; and hemoglobin ≥ 8.0 g/dL). In addition, patients were considered candidates for renal biopsy only if they were not hypertensive (with a blood pressure < 140 × 90 mmHg) and were not using anticoagulant or antiplatelet medications.

The data collected were exported to a Microsoft Excel database. The descriptive analysis was based on the calculation of the absolute and relative frequencies of the variables studied.

## RESULTS

We collected data for 97 renal biopsies, of which 5 (5.2%) were excluded because they were repeat biopsies. Of the 92 remaining biopsies, 87 (94.6%) produced a definitive histopathological diagnosis, thus being considered appropriate, and 5 (5.4%) were inconclusive, thus requiring a new procedure. The mean number of fragments collected per procedure was 2.42, and the mean number of glomeruli per sample was 14.89 (range, 0-55).

The mean age of the 92 patients whose biopsies were evaluated in the present study was 36.2 ± 16.0 years (range, 13-84 years). Less than half (38.0%) of the patients were male, with a mean age of 37.1 ± 16.9 years (range, 13-83 years). Despite being predominant in the sample, the females had a mean age of 35.6 years ± 16.3 years (range, 13-84 years), comparable to that of the males.

In our sample, the main indication for renal biopsy was nephrotic syndrome (in 50.0%), followed by acute kidney injury/rapidly progressive glomerulonephritis (in 19.6%), nephritic syndrome (in 13.0%), chronic kidney disease (in 9.8%), and urinary anomalies (in 9.8%). Among the 46 cases of nephrotic syndrome, the main pathological findings were segmental/focal glomerulosclerosis and lupus nephritis, which were diagnosed in 14 and 13 cases, respectively. Among the specific diagnoses found, the most prevalent was lupus nephritis, which was diagnosed in 32 biopsies (36.8%). The remaining diagnoses are shown, in descending order, in Table 1.

**Table 1:** Pathological results of representative renal biopsies (n = 87).

Primary diagnosis	N	%
Lupus nephritis	32	36.8
Focal segmental glomerulosclerosis	18	20.7
Membranous glomerulonephritis	8	9.2
IgA nephropathy	6	6.9
Amyloidosis	5	5.7
Chronic glomerulopathy	4	4.6
Post-infectious glomerulonephritis	4	4.6
Hypertensive nephrosclerosis	3	3.4
Diabetic nephropathy	2	2.3
ANCA-associated vasculitis	2	2.3
Acute interstitial nephritis	1	1.1
Acute tubular necrosis	1	1.1
Membranoproliferative glomerulonephritis	1	1.1

ANCA, anti-neutrophil cytoplasmic autoantibodies

Immediate complications related to the procedure occurred in seven patients, manifesting as macroscopic hematuria in three (3.3%), perirenal bleeding in three (3.3%), and subcapsular bleeding in one (1.1%). Follow-up imaging evaluations (ultrasound or CT) were performed in patients who evolved to worsening of red blood cell counts, hemodynamic instability, flank pain, or macroscopic hematuria. There was no procedure-related death or report of late complications in patients who were followed at the outpatient nephrology clinic.

**Figure 5.**
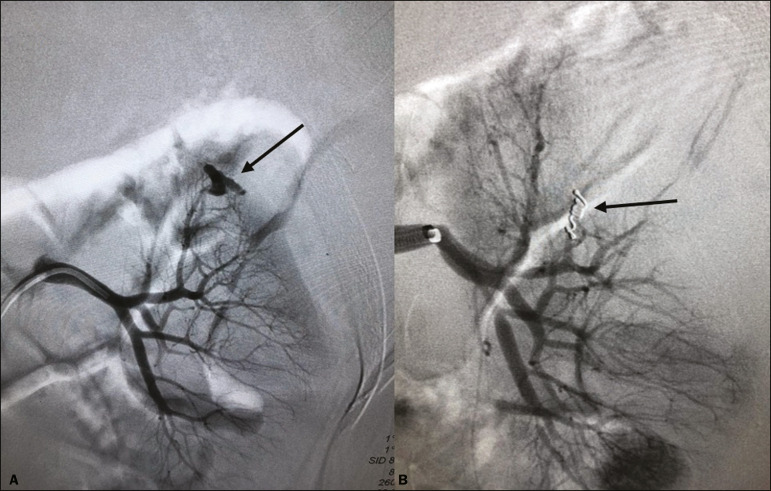
**A:** Selective arteriography of the left kidney, showing a pseudoaneurysm resulting from puncture of the upper pole of the left kidney. **B:** Selective postembolization arteriography, with a 2 mm × 2 cm controlled-release fibered coil, showing occlusion of the puncture pseudoaneurysm.

Of the four patients who had hemorrhaging after the procedure, three (3.3% of the sample as a whole) eventually required emergency clinical intervention. Two patients received blood products and, among the procedures performed, surgery for hemostasis and arteriography with embolization (Figure 5) were necessary in one and two patients, respectively. None of the patients underwent nephrectomy.

## DISCUSSION

Image-guided biopsies have been evaluated in a number of recent articles in the radiology literature of Brazil^(9-14)^. Ultrasound-guided percutaneous renal biopsy is established as a safe routine procedure, with a well-defined risk profile and a low complication rate^(15)^. Studies conducted at several referral centers have evaluated the application of this type of biopsy, involving real-time ultrasound and an automated biopsy device, and have shown that it is associated with low complication rates^(16)^.

The size of our sample was small in comparison with the samples evaluated in other studies^(17-19)^, which can be explained by the fact that, at the facility where our study was conducted, the interventional radiology department and the outpatient clinic for nephrology and glomerular diseases were established only recently (in March 2017).

In the present study, the success rate for obtaining adequate samples by needle biopsy was 94.6%, which is lower than the 100.0%, 99.0%, and 97.5% reported by Manno et al.^(17)^, Korbet et al.^(18)^, and Muñoz et al.^(19)^, respectively. However, the repetition of the procedure in inconclusive cases increased our success rate to 98.9%, corroborating the findings of other authors, who have reported that diagnostic confirmation is achieved in 80-100% of cases^(20)^. The mean of 14.89 glomeruli per sample in our study was adequate and is in accordance with much of the literature, in which approximately 10 glomeruli per sample is considered appropriate^(18,21)^.

A small portion of the patients in our sample (7.6%) experienced complications inherent to the procedure, which is a promising result, despite the fact that interventional radiology is in the incipient phase at our facility, when compared with the complication rates reported in other studies. However, that result should be analyzed with caution, because follow-up imaging exams (ultrasound or CT) were performed only in cases in which there were clinical repercussions, such as macroscopic hematuria, flank pain, worsening of red blood cell counts, and hemodynamic instability. Therefore, it is possible that complications with less pronounced clinical or biochemical features were not identified. In a study conducted by Azmat et al.^(22)^, involving 220 patients, complications occurred in 42 patients (19.1%), major complications, including hypotension and a significant hematoma requiring blood transfusion, occurring in 16 patients (7.3%) and minor complications, including macroscopic hematuria and renal hematoma < 5 cm, occurring in 26 (11.8%).

Tang et al.^(23)^ evaluated 203 ultrasound-guided percutaneous renal biopsies and reported a complication rate of approximately 8%, which is similar to the proportion observed in our study, although our sample was smaller. The Tang et al.^(23)^ study was also limited by the fact that post-procedure ultrasound was used only in patients who presented changes in clinical parameters (e.g., tachycardia or macroscopic hematuria). Those authors reported that most of complications were minor, mild hematuria occurring in 4.5% of the cases, whereas only 1.5% presented major complications that required blood transfusion and only 1.0% required percutaneous intervention with embolization.

In another study of percutaneous renal biopsies, Guerrero-Ramos et al.^(24)^ reported a complication rate of 5.6%, an interventional approach being required in only 1.67% of the cases. Their findings are similar to those of Ali et al.^(25)^, who evaluated 527 ultrasound-guided or “hands free” (ultrasound-assisted) renal biopsies, reporting an overall complication rate of 5.64% and a 2.84% rate of major complications. In the Ali et al.^(25)^ study, the use of a 14-gauge needle was identified as a risk factor for complications in the ultrasound-assisted biopsies, whereas there was no statistically significant difference in risk between the use of a 14-gauge needle and that of a 16-gauge needle (as used in our study) in the real-time ultrasound-guided biopsies. In that study, there were 213 patients who underwent ultrasound-guided biopsy with a 16-gauge needle, of whom two (1.29%) required blood transfusion and one (0.60%) required arterial embolization. In our study, only 16-gauge needles were used, the rate of hemorrhagic complications was 4.3%, two patients requiring blood transfusion and two requiring arterial embolization.

Among the 85 biopsies in which the pathological finding was glomerulopathy in our sample, there was a predominance of secondary glomerulopathies over primary glomerulopathies, which accounted for 56.5% and 43.5%, respectively. The most common pathological diagnosis (seen in 36.8% of the biopsies) was lupus nephritis, followed by focal segmental glomerulosclerosis (in 20.7%), and membranous glomerulonephritis (in 9.2%). Our results are similar to those reported by Muñoz et al.^(19)^, who observed lupus nephritis in 44.7% of cases, focal segmental glomerulosclerosis in 16.4%, and membranous nephropathy in 9.3%. Our findings are also similar to those found in the Pernambuco State Registry of Glomerulopathies^(26)^, which included biopsies performed in the state capital (Recife) between 1998 and 2016, as well as being similar to those of a study conducted in the south of Brazil^(21)^.

Our study has some limitations. Because our study was based on information collected from electronic medical records, we faced challenges related to missing data and poorly organized archives, as well as difficulty in accessing the results of some previous laboratory tests, which are common limitations of retrospective studies. The small number of samples in our study is another limiting factor. However, the results obtained are relevant, given that the study was conducted at a facility that was established only a few years ago and in a semi-arid region of Brazil (the São Francisco Valley). In addition, our data may be included in the Pernambuco Registry of Glomerulopathies and serve to improve understanding of the behavior of glomerulopathies in this region of the country.

## CONCLUSION

Ultrasound-guided biopsy has proven to be a safe, effective method for the diagnosis of nephropathies, especially glomerulopathies, with a high rate of success in making a specific diagnosis. In addition, the success rates are even higher when repeat biopsies are performed.

In our sample, the main indication for biopsy was nephrotic syndrome and the most prevalent pathological diagnosis was lupus nephritis. The procedure presented a low complication rate and there were no instances of nephrectomy or death as a result of the procedure.
